# Extramammary Paget disease of the vulva: immunohistochemical analysis of neoangiogenesis and epithelial-mesenchymal transition markers expression

**DOI:** 10.1186/s13000-017-0680-x

**Published:** 2018-01-03

**Authors:** Lara Alessandrini, Nicolò Clemente, Tiziana Perin, Giorgio Giorda, Vincenzo Canzonieri, Francesco Sopracordevole

**Affiliations:** 1 Pathology Unit - CRO - IRCCS, National Cancer Institute, Aviano, Italy; 2 Gynecological Oncology Unit - CRO - IRCCS, National Cancer Institute, Aviano, Italy; 3 Pathology Unit - CRO - IRCCS, National Cancer Institute, Via Franco Gallini, 2, I-33081 Aviano, (PN) Italy

**Keywords:** Paget disease, Vulva, Neoangiogenesis, EMT, Microvessel density, HVD, AVD

## Abstract

**Background:**

Extra-mammary Paget’s disease of the vulva (EMPDV) is an infrequent chronic disease that often recurs. The aim of the study was to assess the presence of neoangiogenesis and the expression of epithelial-mesenchymal transition (EMT) markers in EMPDV, and their potential correlation with stromal invasion.

**Methods:**

All the women consecutively treated for EMPDV at our Institute, between January 2011 and December 2014, were studied for neoangiogenesis, analysed by microvessel density (MVD) using antibodies against CD31 and CD34. Immunohistochemical expression of E- / N-cadherin, β-catenin and SLUG was also evaluated. In each slide, three fields with the highest number of capillaries and small venules were identified at low power. In these three fields, the highest vessel density (HVD) and the average vessel density (AVD) at 200× and 400× magnification were counted. Immunohistochemical reactions for non-vascular markers were semiquantitatively scored by two pathologists, using a three-tier scale.

**Results:**

Seventeen cases of EMPDV (including 10 cases of invasive disease) were included. The AVD at 200× and 400× and the HVD at 400× magnification were significantly associated with invasive EMPDV (*p* = 0.02, 0.03, 0.03 respectively). No significant correlation between MVD, EMT-markers expression and risk of recurrence was detected.

**Conclusion:**

These results indicate that MVD, as a measure of neoangiogenesis, may be associated with histological progression of EMPDV. EMT could also be linked to an invasive potential of EMPDV but larger series are required to confirm this hypothesis.

## Background

Extra-mammary Paget’s disease of the vulva (EMPDV) is an infrequent chronic disease that often recurs and accounts for less than 1% of vulvar neoplasms [1]. It is characterized by peculiar cells (so-called “Paget cells”) with pale large cytoplasm and prominent nucleoli, that are located in single cells or in clusters throughout the epithelium, and may form gland-like structures [2]. In the pathologic report of EMPDV, it is mandatory to evaluate the presence/absence of stromal invasion, which is defined by the occurrence of dyscohesive neoplastic cells infiltrating the underlying dermis or submucosa and to distinguish between superficially invasive carcinoma (stromal invasion ≤1 mm, according to the International Federation of Gynecology and Obstetrics [FIGO] stage IA) [3] and invasive carcinoma. Many clinical and pathological aspects of EMPDV are debated in Literature: the disease progression seems to be influenced by site, presence of invasion, and extent of the surgical treatment. As microscopic disease often extends beyond the clinically visible lesion, surgical excision, which remains the treatment of choice, cannot prevent the frequent recurrences of EMPDV [4].

Recent data have shown that disease course may be influenced by some tissue markers related to tumor angiogenesis and epithelial/mesenchymal transition. Tumor angiogenesis is the rise of new blood vessels from pre-existing vasculature: this complex event is crucial in tumor growth and metastasis [5]. Microvessel density (MVD) could be used to evaluate angiogenesis by a semi-quantitative method. An increased MVD has been found in various tumors, including vulvar carcinoma [6, 7], and has been associated with disease progression and development of metastasis. Different endothelial cell markers have been used to identify microvessels, including CD31 and CD34. CD34 is a 110-kD protein expressed by embryonic cells of the hematopoietic system and also by endothelial cells [8]. CD31 is a 130-kD transmembrane glycoprotein that is commonly expressed by vascular lining cells, platelets, and other hematopoietic elements [8].

In the last years, the “epithelial–mesenchymal transition” (EMT), a key developmental regulatory program, has been reported to promote tumor invasion and metastasis in epithelium-derived tumors [9]. A hallmark of EMT is the decreased expression of CDH1 (E-cadherin) followed by increased CDH2 and/ or CDH3 expression (respectively, N- and P-cadherin), a process called “cadherin switching” [10].

Moreover, several transcription factors (including Snail-related zinc-finger transcription factors - Snail and Slug -), have been found to trigger the down-regulation of E-cadherin and, consequently, to produce EMT in different types of cancer [11]. Cadherins are calcium-dependent transmembranous intercellular adhesion molecules, which have distinctive immunologic specificities and tissue distributions. They are involved in selective cellular adhesion and are typically associated with epithelial differentiation [11]. Altered E-cadherin expression has already been documented in EMPDV [12] and vulvar intraepithelial neoplasm [13]. β-catenin is a closely related protein that is involved in cell adhesion (adherens junctions), and in the Wnt signalling pathways, an important pathway in cellular development and cancer progression [14].

During EMT, loss of E-cadherin and altered regulation of β-catenin occur. As a consequence, β-catenin tends to localize in the nucleus, where it acts as a co-transcriptional regulator, contributing to the transcriptional activation and increased expression of mesenchymal markers and indirectly influencing further down-regulation of epithelial markers, such as E-cadherin [14].

The aim of this study was to evaluate the presence of neoangiogenesis and the expression of EMT-related markers in EMPDV and their potential correlation with stromal invasion, verifying whether there is different expression of these markers and of angiogenesis in invasive versus non invasive EMPDV.

## Methods

### Patients’ selection

All the women consecutively treated for EMPDV at our Institute, between January 2011 and December 2014, were considered. In each patient, the diagnosis of EMPDV was initially obtained on vulvar biopsies and then confirmed on final surgical specimens obtained with wide vulvar resection or skinning vulvectomy/hemivulvectomy.

Only the women at their first diagnosis of EMPDV were considered, thus women who underwent surgery for a recurrence of the disease were excluded. Similarly, women with a concomitant diagnosis of vulvar or vaginal high grade squamous intraepithelial neoplasia and/or invasive vulvar or vaginal cancer on the surgical specimen were excluded.

Only women with a follow up of at least 24 months were included in the present analysis.

All the women fulfilling the study inclusion/exclusion criteria were retrospectively identified through a search of our institutional databases. The medical charts of women included in the present study were then reviewed, and pertinent clinical and histopathological data were collected. More in detail, depth of invasion was evaluated on hematoxilyn & eosin- stained slides, using an ocular micrometer to measure the depth of invasion from the surface of the epidermis or squamous epithelium to the deepest tumor cells in the dermis.

The “persistence” of the lesion was defined as the histopathological diagnosis (through vulvar biopsy) of EMPDV at the first follow-up gynecological examination (performed 3–6 months after the surgical excision). The “recurrence” was defined as the histopathological diagnosis (through vulvar biopsy) of EMPDV after at least one negative follow-up gynecological examination.

### Immunohistochemical analyses

All the immunohistochemical analyses were performed on the final surgical specimens (wide vulvar resection or skinning vulvectomy/hemivulvectomy). For all patients, both previous pathologic reports and slides were independently reviewed by two pathologists (VC, LA). 2.5-μm sections were cut from formalin-fixed paraffin embedded (FFPE) tissue of each patient and immunohistochemical analysis was performed in an automated system (Benchmark-Ultra, Ventana, Tucson, AZ, US). The following primary antibodies were used: CD34 (monoclonal antibody, clone QBEND/10; 1:400 dilution; Neomarkers, Freemont, CA, USA), CD31 (monoclonal antibody, clone JC70, Prediluted, Cell Marque, Rocklin, CA, US), E-cadherin (monoclonal antibody, clone 36, pre-diluted, Ventana, Tucson, AZ, US), N –cadherin (monoclonal antibody, clone 6G11, dilution 1:50, Dako, Glostrup, Denmark); SLUG (monoclonal antibody, 1A6, dilution 1:100, Novus Biologicals, Littleton, CO, USA), β-catenin (monoclonal antibody, clone 14, ready to use, Cell Marque).

Normal vulvar skin was used as positive control while for negative controls, the primary antibodies were replaced by PBS.

### Immunohistochemistry evaluation

To evaluate MVD, in each slide a dermal area 500 μm beneath the basement membrane was selected. CD34 and CD31 antibodies displayed overlapping staining characteristics. Countable microvessels were defined as brown staining endothelial cell clusters that were separated from each other, as reported elsewhere [15, 16]. Large vessels were excluded from the count. In each slide, three fields with the highest number of capillaries and small venules were identified at low power. In these three fields, the highest vessel density (HVD) and the average vessel density (AVD) at 200× (0.950 mm^2^/field under the light microscope) and 400× (0.237 mm^2^/field under the light microscope) magnification were counted. Immunohistochemical reactions for non-vascular markers were semiquantitatively scored, using a three-tier scale as follows: - For E-cadherin, N-cadherin and β-catenin, a membranous staining in >90% of the Paget cells was scored 2, a heterogenous staining (between 10% and 90% of the cells with membranous staining) was scored 1 and membranous staining in <10% of the cells was scored as 0, as reported elsewhere [17]. The presence of a different cellular pattern of expression for these markers was also considered: cytoplasmic staining for E-cadherin and N-cadherin and nuclear staining for β-catenin. - For SLUG, 0% of positive cancer cells were scored 0; 1–49% of nuclear positive cancer cells were scored 1; 50–70% of nuclear positive cancer cells were scored 2 and 3 > 70% of positive cancer cells [18].

Sections were stained on two distinct occasions and scored separately by two independent pathologists (VC, LA) to ensure reproducibility.

### Statistical analysis

Statistical software SPSS 20 (SPSS Inc., Chicago, IL, USA) was used for data analysis. All continuous variables were tested for normality with the D’Agostino-Pearson test. Normally distributed variables were expressed as mean ± SD. The t-test was used for comparison as appropriate. Qualitative variables were expressed as proportions and were compared with Chi-square or Fisher’s exact test as appropriate. A *P* value of <0.05 was considered statistically significant.

## Results

### Patients

Seventeen cases of EMPDV, fulfilling the study inclusion/exclusion criteria were considered for the present analysis. All the women included were post-menopausal, and the mean age (± SD) was 69.5 ± 8.7 years (range: 53–84 years). The main clinical and pathological characteristics of the study cohort are reported in Table 1.

**Table 1:** Clinical and pathological characteristics of women of the study cohort

Patient	Age	Type of surgery	Stromal invasion	FIGO stage	LVSI	Distance of closest margin	Persistence	Recurrence
1	63	Wide local excision	< 1 mm	IA	absent	< 10 mm	No	Yes (24 months)
2	71	Wide local excision	< 1 mm	IA	absent	0 mm	No	No (31 months)
3	72	Wide local excision	absent	–	absent	< 10 mm	No	Yes (14 months)
4	71	Skinning vulvectomy	absent	–	absent	<1 mm	No	No
5	62	Skinning vulvectomy + LNF	absent	–	absent	0 mm	No	Yes (12 months)
6	74	Skinning vulvectomy	absent	–	absent	< 1 mm	No	Yes (36 months)
7	75	Wide local excision	< 1 mm	IA	absent	0 mm	No	No
8	80	Skinning vulvectomy	absent	–	absent	< 10 mm	No	Yes (14 months)
9	53	Skinning vulvectomy	< 1 mm	IA	absent	0 mm	No	Yes (31 months)
10	72	Skinning vulvectomy	> 1 mm	IB	absent	0 mm	No	No
11	84	Wide local excision	< 1 mm	IA	absent	< 1 mm	No	No
12	59	Skinning vulvectomy	< 1 mm	IA	present	0 mm	No	Yes (14 months)
13	58	Wide local excision	absent	–	absent	0 mm	Yes	–
14	67	Skinning vulvectomy	< 1 mm	IA	absent	0 mm	No	No
15	73	Wide local excision	< 1 mm	IA	absent	0 mm	No	No
16	65	Wide local excision	absent	–	absent	< 1 mm	No	No
17	83	Skinning vulvectomy + LNF	> 1 mm	IB	present	0 mm	Yes	No

*LVSI* lymphovascular space invasion

**Fig. 1:**
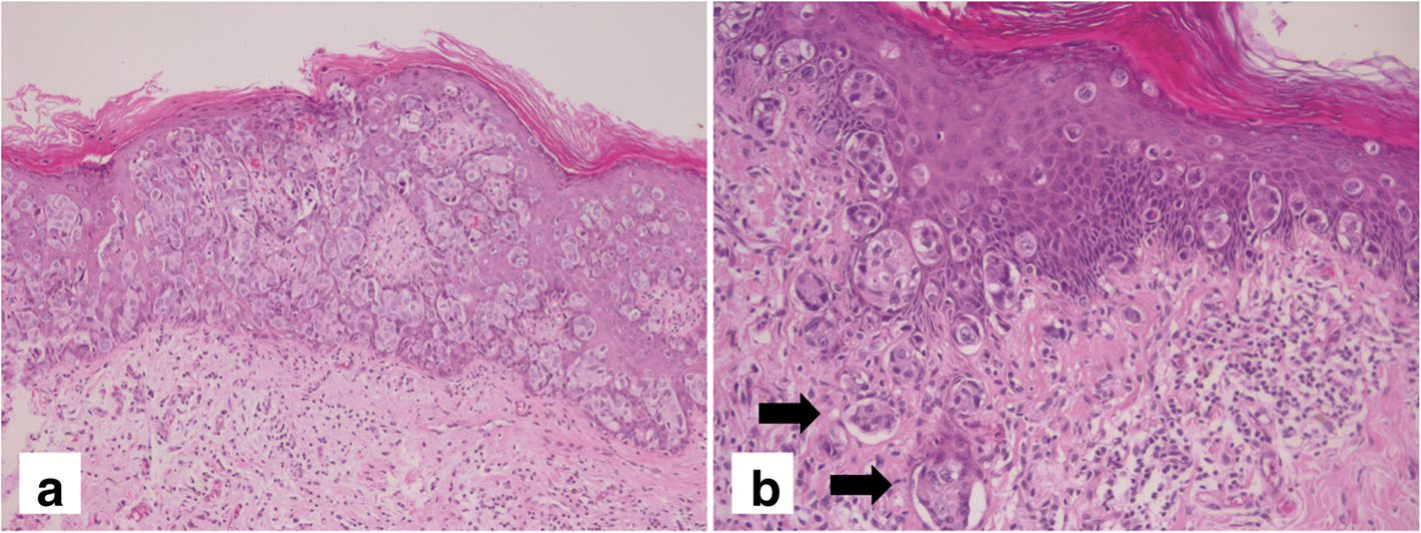
**a**, **b**. Large cells (“Paget cells”) with pale cytoplasm and large nuclei with prominent nucleoli, arranged either singly or in clusters throughout the epithelium. **b**. Stromal invasion and lymphovascular space invasion (arrows) are evident (Hematoxylin & eosin; A: original magnification ×200; B: original magnification ×400)

All resection specimens had negative surgical margins on macroscopic evaluation, with a distance between the visible margins of the lesion and the resection margins ranging from 5 to 20 mm. However, surgical margins were microscopically positive for neoplasia in 10 cases (58.8). Stromal invasion was present in 10 out of 17 cases (58.8%): 8 cases were superficially invasive carcinomas (FIGO stage IA) with less than 1 mm of invasion (Fig. 1), and two patients had FIGO stage IB lesions.

### Immunohistochemistry

Immunohistochemical scores for each patient (in the final surgical specimen) are reported in Table 2. The mean (± SD) HVD at 200× and 400× magnification in EMPDV without invasive disease were 30.9 (± 14.5) and 14.1 (± 5.9), respectively, whereas in invasive EMPDV, the mean (± SD) HVD at 200× and 400× magnification were 41.3 (± 20.8) and 23.7 (± 9.5), respectively.

**Table 2:** Immunohistochemical findings of MVD and EMT- related markers

Patient	HVD (200x)	HVD (400x)	AVD (200x)	AVD (400x)	E-cadherin	N-cadherin	β-catenin	Slug
1 ^a^	1	16	25,00	12,67	2	1	1	0
2 ^a^	32	14	25,34	12,00	2	0	1	0
3	16	10	12,00	0,00	2	1	0	0
4	57	26	38,34	17,34	2	0	1	1
5	40	16	22,34	11,34	2	0	0	0
6	21	12	18,00	8,67	2	0	1	0
7 ^a^	57	28	43,67	22,00	2	1	1	2
8	24	11	16,67	7,67	2	0	1	1
9 ^a^	22	13	19,67	11,34	2	0	1	2
10 ^a^	30	12	22,00	10,00	2	1	1	0
11 ^a^	54	33	41,67	21,34	2	1	1	0
12 ^a^	50	29	47,67	25,00	2	1	1	0
13	21	11	18,67	20,00	2	2	1	0
14 ^a^	37	22	35,67	28,00	2	2	1	1
15 ^a^	60	32	54,67	28,00	2	1	1	0
16	37	13	27,67	13,34	2	0	1	1
17 ^a^	70	38	63,67	29,67	2	0	1	0

^a^ patients with invasive disease

*MVD* microvessel density, *HVD* highest vessel density, *AVD* average vessel density, *EMT* epithelial-mesenchymal transition

**Fig. 2:**
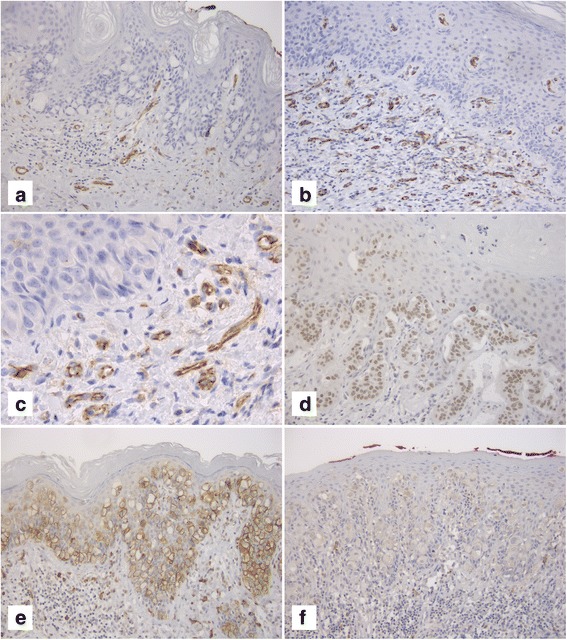
**a** A case with a low value of HVD (microvessels are highlighted by CD31 immunostaining) (original magnification ×200); **b** A case with a high value of HVD (microvessels are highlighted by CD31 immunostaining) (original magnification ×200); **c** Same case as B (original magnification ×400); **d** Paget cells show positive nuclear staining for SLUG; **e** Positive immunostaining for N-cadherin in Paget cells (original magnification ×200); **f** Negative immunostaining for N-cadherin in Paget cells (original magnification ×200)

Therefore, HVD at 400× was significantly higher in women with invasive EMPDV (23.7 ± 9.5 vs 14.1 ± 5.9; *P* = 0.03), while HVD at 200× was higher, but the difference was not statistically significant (41.3 ± 20.8 vs 30.9 ± 14.5; *P* = 0.3) (Fig.2A,2B,2C).

The mean AVD (± SD) at 200× and 400× magnification in EMPDV without invasive disease were 21.9 (± 8.7) and 11.2 (± 6.6) respectively, whereas in invasive EMPDV, the mean (± SD) AVD at 200X and 400X magnification were 37.9 (± 14.9) and 20 (± 7.8). Therefore, AVD at 200X and 400X were both higher in invasive EMPDV compared to non-invasive EMPDV (37.9 ± 14.9 vs 21.9 ± 8.7; *P* = 0.02 and 20 ± 7.8 vs 11.2 ± 6.6; *P* = 0.03, respectively).

The immunohistochemical scores for E-cadherin were the same in women with invasive EMPDV compared to non-invasive EMPDV and only membranous staining was observed. Positive immunostainings for N-cadherin were observed in 7 cases with invasive EMPDV (70%) and in 2 cases with non-invasive EMPDV (28.6%), but the difference was not significant (*P* = 0.2) (Fig.2E,2F). Positive membranous staining for β-catenin was observed in all the cases with invasive EMPDV and in 5 cases with non-invasive EMPDV (71.4%), and even in this case the difference was not significant (*P* = 0.3). No nuclear staining for β-catenin was found.

Similarly, comparing the cases with invasive EMPDV to non-invasive EMPDV, no differences were observed about SLUG positivity immunostaining (30% vs 42.9%, *P* = 0.9) (Fig.2D).

### Persistence/recurrence

Among the 17 women included in the present analysis, only one showed a persistence (as previously defined), while eight women had a recurrence (47%). In these patients the mean time to recurrence was 22 months. On a multivariable logistic regression, no one of the potentially involved factors (positive surgical margins, stromal invasion and LVSI) was associated to the risk of recurrence. Similarly, no one of the evaluated immunohistochemical markers of neoangiogenesis and EMT showed a correlation with the risk of recurrence.

## Discussion

Extramammary Paget’s disease is an unusual skin neoplasm with unclear pathogenesis [19]. The most common site of involvement is the vulva [19] but, because of its rarity (1%–2% of vulvar malignant tumors), its true incidence and prevalence remains unclear [20, 21].

The Paget cells can present as single cells or nests in the epithelium of squamous mucosa or adnexa. Their spread can also affect areas of apparently healthy skin, and the disease can microscopically extend beyond the clinical apparent edges of the lesion [19].

Moreover, the currently used staging system of the vulvar cancer [3] seems to be inadequate to guide the treatment choices of a neoplasia characterized by diffuse superficial extension and limited invasion (often less than 1 mm) [19].

EMPDV can be considered a chronic disease with a high risk of relapse, even many years after the initial diagnosis; however, the risk factors for recurrence are still unclear [19]. In particular, several studies have reported no apparent correlation between surgical margin status and disease recurrence [19, 22–24]. More in detail, Shaco-Levy et al. [22] showed that recurrences occurred more frequently following resections with positive permanent margins, but this relation was not statistically significant (*p* = 0.14). Additionally, a recent review compared studies that looked at associations between recurrence rates and surgical margin status. According to Literature, the influence of surgical margin status on recurrence rates showed contrasting results [25].

Notably, even in our series, microscopically positive surgical margins, observed in 58,8% of cases, seem not to be associated with significant high risk of recurrence. However, it is important to underline that a high percentage of cases without macroscopic neoplastic involvement of surgical margins, have microscopic positive margins. EMPDV act as a slowly progressive disease [19], but may acquire an aggressive phenotype when there is deep neoplastic stromal invasion, with a higher risk of distant metastasis and mortality [26].

For this reason, it is mandatory to get a precise histopathological diagnosis of stromal invasion, in order to identify those women who may require more extensive surgery and a closer post-treatment follow up.

In order to refine the prognostic/predictive value of morphological and phenotypic features in EMPDV, we considered neoangiogenesis (analyzed through MVD) and EMT markers expression in the development of invasive disease. The role of angiogenesis, as determined by MVD, has already been examined in vulvar intraepithelial neoplasia (VIN), and vulvar cancer [7, 27, 28]. Increased MVD was associated with progression to invasive disease in VIN3 cases [7], and with a poor prognosis in squamous cell carcinoma (SCC) of the vulva [27, 28].

In our study, we found a significant association between higher MVD and the presence of invasive EMPDV: AVD at 200× and 400× and HVD at 400× were significantly associated with invasive EMPDV (*p* = 0.02, 0.03, 0.03 respectively) when compared with non invasive disease. These findings suggest that neovascularization is an important factor in the development of invasion in EMPDV. However, our results are in contrast with those of the largest study investigating MVD in EMPDV currently available [16], that demonstrated an increased MVD in Paget’s disease of the breast with DCIS/infiltrating carcinoma (23 cases) compared to Paget’s disease of the breast alone (11 cases)(*p <* 0*.*08 and *p <* 0*.*013, respectively), whereas no significant differences in MVD in vulvar Paget’s disease cases, both invasive (8 cases) and non-invasive (63 cases) were found.

Several reasons could explain these contrasting results. First, we used different antibodies (CD31 and CD34 instead of vWF) to stain microvessels and we did not find unspecific staining in other tissue components; second, we cut sections from FFPE tissues dating from 6 to 3 years ago, having probably less influence on antigen/epitope retrieval; third, we performed immunohistochemical analyses using and automated system and stained and evaluated sections on two separate occasions by two independent pathologists to ensure reproducibility.

At a cellular level, during the neoangiogenetic process, vascular buds are made of distinctly differentiated endothelial cell subtypes [29], including endothelial tip cells. These cells form filopodia to aid migration towards a source of growth factors (such as vascular endothelial growth factors [VEGF]); and to direct adjacent endothelial cells to elongate the stalk of newly developed vessels [29]. Endothelial tip cells and their filopodia [29] highly express CD34, suggesting a role for CD34 in angiogenesis, specifically related to filopodia functions or architecture.

Interestingly, in a recent paper [30], the expression of pro-angiogenetic factors, such as VEGF, have been significantly more evident in extramammary Paget disease compared with normal tissue. The identification of targeted drugs that can block blood supply of tumor cells could widen the spectrum of the treatment options for EMPDV.

Our preliminary data identify a higher MVD in patients with invasive EMPDV. If validated in larger series, the measure of MVD may play a role in predicting invasion in small biopsies with Paget disease, helping to plan a more appropriate treatment and follow-up.

Currently, there are no treatment guidelines for EMPDV, but surgery is considered the cornerstone of treatment [31]. However, because of the extension and the frequent multifocality of the lesion, surgical excision can cause significant vulvar mutilation and several complications [19, 25]. Moreover, surgery is not always possible due to the location or size of the lesions or because of patients’ characteristics and preferences [25]. Therefore, a pressing need for alternative treatments is emerging [25]. Several non-surgical therapies have been proposed for EMPDV, such as topical imiquimod cream, radiotherapy or photodynamic therapy (PDT).

Theoretically, the identification of immunohistochemical markers of invasive disease (such as MVD) on biopsy could be useful to differentiate women who are more likely to have an invasive disease (and thus could benefit from a more radical surgical intervention) and women with a lower risk of invasive disease. These patients could therefore be referred to non-surgical therapies. However, the efficacy of such therapies is currently on debate and, even if some interesting results have been reported, they are limited to small series, and further studies on larger samples are needed to define the real efficacy of such therapies compared to surgery.

Since the loss of expression of E-cadherin [12–14] and the altered β-catenin expression [12, 14] have been suggested as one of the mechanisms contributing to development of invasion in EMPDV, we also have investigated their expression with a view to correlating lack of expression with invasive disease. Intriguingly, no altered expression of these proteins and the other EMT markers, N-cadherin and Slug, was found in our series, therefore further studies on larger series are advisable to explore the potential correlation of these markers with the development of invasive EMPDV.

## Conclusions

MVD, as a measure of neoangiogenesis, may be associated with histological progression of EMPDV, since it seems to be related to presence of invasive disease. Higher MVD identifies a subset of women who may require more accurate controls. If validated in larger series, the measure of MDV may play a role in predicting invasion in small biopsies with Paget disease, helping to plan a more appropriate treatment and follow-up. Further studies are needed to verify this hypothesis and to identify antiangiogenic drugs, as a possible treatment in high-risk EMPDV.

## Abbreviations

*AVD*: Average vessel density
*EMPDV*: Extra-mammary Paget’s disease of the vulva
*EMT*: Epithelial/mesenchymal transition
*HVD*: Highest vessel density
*MVD*: Microvessel density
